# Learning Curve and Video-Based Technical Assessment of a Standardized Vesicourethral Anastomosis in Novice Robotic Surgeons

**DOI:** 10.3390/curroncol33090502

**Published:** 2026-08-25

**Authors:** Federico Germinale, Manfredi Bruno Sequi, Antonia Di Domenico, Andrea Benelli, Federico Dotta, Marco Ennas, Giovanni Guano, Claudia Brusasco, Mattia Tosi, Martina Manfredi, Antonio Luigi Pastore, Antonio Carbone, Andrea Fuschi, Carlo Introini

**Affiliations:** 1 Urology Unit, E.O. Ospedali Galliera, 16128 Genoa, Italy; 2 Urology Unit, Department of Medico-Surgical Sciences & Biotechnologies, Faculty of Pharmacy & Medicine, Sapienza University of Rome, 04100 Latina, Italyantonioluigi.pastore@uniroma1.it (A.L.P.);; 3 Anaesthesia and Intensive Care Unit, E.O. Ospedali Galliera, 16128 Genoa, Italy

**Keywords:** robot-assisted radical prostatectomy, vesicourethral anastomosis, learning curve, robotic surgery training, video-based assessment, surgical education, urinary continence

## Abstract

**Background/Objectives**: Vesicourethral anastomosis (VUA) is one of the most technically demanding steps of robot-assisted radical prostatectomy (RARP) and may influence perioperative outcomes and early urinary continence recovery. Objective assessment of technical performance during the learning curve remains limited. We evaluated the learning curve and technical performance of a standardized VUA technique among novice robotic surgeons using a structured video-based assessment. **Methods**: In this retrospective single-center study, 100 consecutive patients undergoing RARP performed by four novice robotic surgeons were analyzed. All surgeons used the same standardized VUA technique. Operative videos were reviewed and assessed using a modified Robotic Anastomosis Competency Evaluation (RACE) score. Learning-curve trends were analyzed using LOWESS smoothing and mixed-effects regression models. The primary outcome was VUA time. Secondary outcomes included technical performance, anastomosis-related complications, catheterization duration, and urinary continence recovery. **Results**: A total of 100 procedures were included (25 per surgeon). Increasing surgical experience was significantly associated with shorter VUA time (β = −6.93, 95% CI −7.42 to −6.44; *p* < 0.001) and higher modified RACE scores (β = 4.99, 95% CI 4.55–5.43; *p* < 0.001). Median VUA time decreased from 32 min (IQR 29–37) during the early phase to 19 min (IQR 18–20.3) during the late phase (*p* < 0.001), while median RACE score improved from 13 (IQR 11–15) to 22 (IQR 21–23) (*p* < 0.001). Positive leak tests decreased from 37.5% to 12.5% (*p* = 0.03), and the need for additional stitches decreased significantly (*p* < 0.001). Catheterization time was reduced from 8 to 6 days (*p* < 0.001). Early urinary continence improved from 37.5% to 65% at 30 days (*p* = 0.025) and from 60% to 85% at 90 days (*p* = 0.04), whereas differences were no longer significant at longer follow-up. **Conclusions**: Novice robotic surgeons performing a standardized VUA technique showed progressive improvements in procedural efficiency and technical performance during their early learning curve. These findings describe technical progression within a standardized operative framework but do not establish an independent effect of standardization itself. Enhanced technical proficiency was accompanied by improved perioperative parameters and a faster recovery of early urinary continence. Video-based assessment may represent a valuable tool for objective monitoring of skill acquisition during robotic surgical training.

## Simple Summary

Vesicourethral anastomosis (VUA) is one of the most technically challenging steps of robot-assisted radical prostatectomy, particularly for surgeons early in their robotic experience. In this study, we evaluated the learning curve of four novice robotic surgeons performing the same standardized VUA technique. Video-based assessment showed that increasing surgical experience was associated with shorter anastomosis times and improved technical performance. These changes were accompanied by shorter catheterization and faster early urinary continence recovery, while longer-term continence outcomes were comparable between learning phases. Because the study did not include a non-standardized comparison group, the observed improvements cannot be attributed specifically to technique standardization. Our findings support structured video-based assessment as a useful approach for objectively monitoring technical skill acquisition during robotic surgical training.

## 1. Introduction

Prostate cancer (PCa) represents the most common solid malignancy in developed countries [1,2] and the third cause of death among men in Europe [3]. Robot-assisted radical prostatectomy (RARP) today represents the standard surgical treatment for localized PCa. However, the vesicourethral anastomosis (VUA) remains one of the most technically challenging parts of the procedure. Poorly executed anastomoses can cause urinary leakage, delayed urinary continence (UC) recovery, or late bladder neck strictures (BNSs), all of which directly impact postoperative function and patient quality of life (QoL) [4,5,6]. Evidence indicates that a surgeon’s technical skill influences outcomes, including the expedited recovery of UC following RARP [7]. Novice robotic surgeons may encounter considerable difficulties in mastering the procedure VUA [6]. Learning-curve research shows that multiple procedures are typically required to achieve consistent anastomotic skills and technical proficiency. Although overall surgical durations typically decrease after approximately 10 procedures [8], technique refinement, particularly for steps such as the VUA, tends to progress more gradually [9]. This underscores the need for standardized techniques and objective assessment tools to monitor technical performance during the initial learning period. Standardizing the VUA technique has been proposed to reduce variability and support skill development among novice robotic surgeons [10]. Techniques such as running anastomoses and posterior reconstruction are widely used to enhance reproducibility and improve functional results [11,12]. While clinical outcomes for different VUA techniques have been documented, there is limited objective evaluation of technical performance, particularly during the learning curve [4,6,9,11,12,13,14]. Recently, video-based evaluation has gained recognition as an effective method for objectively assessing surgical skills [7,10]. Reviewing operative videos enables detailed analysis of technical execution and the use of validated scoring systems, such as the Robotic Anastomosis Competency Evaluation (RACE) score [6], which was developed to assess robotic anastomotic skills and distinguish levels of surgical expertise. Still, its use in real-world, learning-curve studies involving novice robotic surgeons has not been thoroughly explored. Thus, we aimed to assess the learning curve and technical performance of four novice robotic surgeons performing a standardized VUA during their early RARP experiences in a single-center retrospective study. By reviewing operative videos and conducting a structured technical assessment using the RACE score, we evaluated changes in anastomosis time, technical quality, anastomosis-related complications, and UC outcomes during follow-up.

## 2. Materials and Methods

### 2.1. Study Design, Surgeon and Patient Selection

We conducted a monocentric retrospective observational study to evaluate the learning curve, technical performance, and functional outcomes of novice robotic surgeons performing a standardized VUA technique during RARP. The study was conducted in accordance with the Declaration of Helsinki and approved by the Institutional Review Board of E.O. Galliera (7/2019 id: 4378, amendment 2 approved on 20 December 2022). All patients included in the study provided written informed consent. All operative recordings were anonymized before analysis, with data management complying with institutional and ethical standards. The focus was on surgeons early in their independent robotic experience, with a video assessment of their technical skills. All surgeons were novices, having performed 10 or fewer independent RARP procedures prior to the first case in the study. None had prior independent experience with the specific VUA technique being evaluated. Procedures were performed at a single institution using a standardized surgical protocol (E.O. Ospedali Galliera, Genoa, Italy). The retrospective review included all consecutive patients undergoing RARP by four novice surgeons from January 2022 to January 2025. Patient assignment was not randomized and was based on the institutional operating schedule and surgeon availability. Cases considered preoperatively to have greater technical complexity were preferentially assigned to experienced robotic surgeons and were therefore not included in the novice-surgeon cohort analyzed in the present study. Exclusion criteria included salvage prostatectomy, a history of pelvic radiotherapy, previous prostatic or peri-prostatic surgery, or major bladder-neck reconstruction. Major bladder-neck reconstruction was defined as extensive remodeling beyond routine bladder-neck tailoring. Preoperative MRI was used to identify median lobes; these cases were assigned to experienced surgeons and excluded from the novice cohort. Accordingly, no median-lobe cases were included (0/25 for each surgeon). Previous prostatic surgery included any prior procedure involving the prostate or bladder neck, such as transurethral resection of the prostate (TURP), holmium laser enucleation of the prostate (HoLEP), open or minimally invasive prostatectomy, transurethral incision of the prostate (TUIP), or other surgical or endoscopic procedures affecting vesicourethral anatomy. Data collected comprised age, BMI, medical history (including diabetes mellitus [DM] and metabolic syndrome), surgical history, PSA, ISUP grade, and prostate volume. Patients with incomplete operative or follow-up data were excluded, and participants were required to have at least 90 days of follow-up.

### 2.2. Surgical Technique

**Figure 1:**
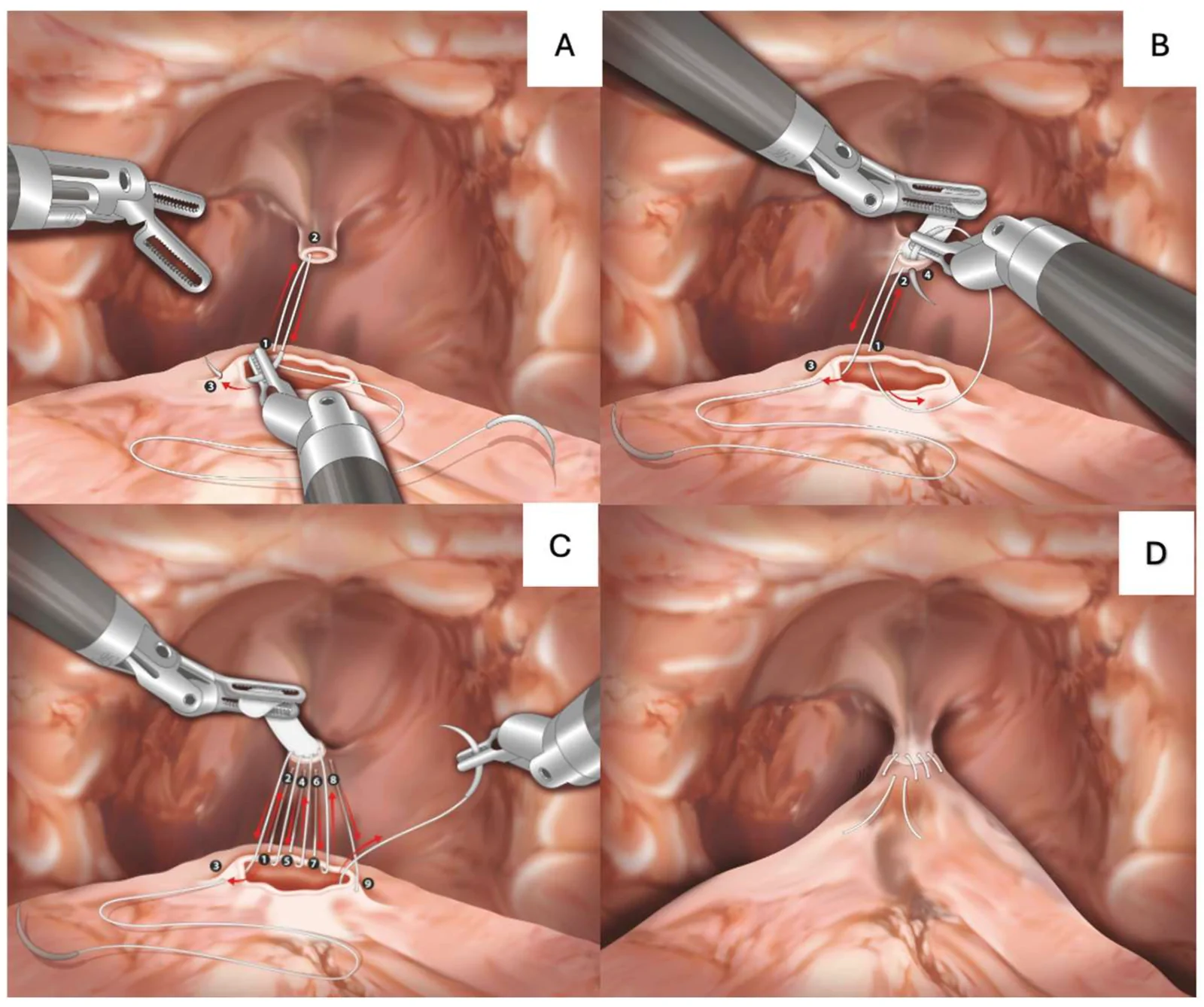
Step-by-step representation of the standardized vesicourethral anastomosis technique using a single double-armed barbed suture. (**A**) Initial stitch placed at the 4 o’clock position on the bladder (inside-to-outside) followed by the corresponding urethral stitch (outside-to-inside). (**B**) Continuation of the suture toward the posterior plate with sequential stitches placed along the bladder and urethral edges. (**C**) Completion of the posterior layer with continuous suturing and approximation of the bladder neck to the urethra. (**D**) Finalization of the anastomosis after catheter insertion, with closure of the anterior plate.

All procedures were performed using the Da Vinci Xi surgical system (Intuitive Surgical Inc., Sunnyvale, CA, USA), in accordance with standard anterior RARP principles. VUA was performed using a predefined, standardized technique in all cases (Figure 1).

The anastomosis is performed using a single double-armed barbed suture (Stratafix 2-0). The procedure begins with a stitch placed on the bladder side at the 4 o’clock position, passing from inside to outside. Using the same needle, a corresponding stitch is then placed on the urethral side at the 4 o’clock position, passing from outside to inside. The needle is then returned to the bladder side, placing another stitch from inside to outside at the 3 o’clock position. At this stage, the second needle on the opposite end of the suture is used to initiate the posterior plate. A stitch is placed on the urethral side at the 5 o’clock position, passing from inside to outside. The posterior layer is then completed using the same needle, proceeding in a continuous fashion with outside-to-inside passes on the bladder and inside-to-outside passes on the urethra. The anastomosis is then continued and completed using only the needle on the left side (i.e., with the dominant hand), progressing toward the anterior plate. After insertion of the definitive urinary catheter, the anastomosis is completed by placing an additional stitch using the same needle.

### 2.3. Operative Video Review and Technical Assessment

All procedures were routinely recorded on the robotic platform. Operative recordings were retrospectively reviewed to accurately determine VUA time and to perform a structured video-based technical assessment. Video segments corresponding to the anastomotic phase were extracted and anonymized. Technical performance was assessed using a predefined adapted version of the RACE score, originally developed and validated for objective assessment of VUA during RARP. Because the original RACE instrument was designed for real-time assessment of supervised trainees, an adapted scoring system was developed a priori for this study to enable standardized retrospective blinded evaluation of independently performed procedures. Compared with the original six-domain RACE score (range 6–30), the adapted version comprised five video-assessable domains: needle positioning and driving; suture handling; tissue handling and respect; symmetry and tissue approximation; and overall anastomotic quality. Knot tying was not retained as a separate domain. Each domain was rated on a five-point Likert scale, yielding a total score ranging from 5 to 25. A detailed description of the adapted scoring system is provided in Supplementary Table S1. Video review was independently performed by two experienced robotic surgeons (C.I. and A.F.) who were not involved in the index procedures. The reviewers were blinded to surgeon identity and case sequence during scoring. Reviewers resolved scoring discrepancies by consensus. Inter-rater reliability was assessed using the intraclass correlation coefficient (ICC). The video of the standardized technique is available as Supplementary Video S1.

The primary outcome was VUA time, defined as the interval between the first needle passage and completion of the VUA, measured using video timestamps. VUA time was selected as the primary outcome because it is an objective, reproducible measure of procedural efficiency. The modified RACE score was considered a key secondary outcome, providing a complementary assessment of technical quality beyond operative time. Other secondary outcomes included anastomosis-related intraoperative and postoperative complications, overall postoperative complications classified according to the Clavien–Dindo classification, and UC. Catheterization duration was determined according to postoperative cystographic assessment of the VUA, using the same catheter management approach throughout the study period.

### 2.4. Learning Curve Definition

Learning-curve analysis was performed in chronological order of procedures for each surgeon. A surgeon-specific case sequence number was assigned according to the order of RARP procedures. For comparative analyses, the first 10 consecutive cases performed by each surgeon were defined as the early phase, and the last 10 consecutive cases as the late phase, yielding 40 procedures in each group. VUA time and modified RACE scores were also analyzed as continuous variables in the order of cases.

### 2.5. Statistical Analysis

Continuous variables were reported as median and interquartile range (IQR) or mean and standard deviation (SD), as appropriate according to data distribution. Categorical variables were expressed as frequencies and percentages. Baseline characteristics were compared among the four surgeons using the Kruskal–Wallis test for continuous variables and the chi-square or Fisher’s exact test for categorical variables, as appropriate, whereas early- and late-phase comparisons were performed using the Mann–Whitney U test and the corresponding tests for categorical variables. Learning-curve trends were explored graphically using LOWESS (locally weighted scatterplot smoothing) to visualize potential non-linear relationships between case sequence and outcomes. To quantify the association between surgical experience and technical performance, mixed-effects linear regression models were fitted. In these models, case sequence was log-transformed and included as a fixed effect, while surgeon was included as a random intercept to account for clustering of procedures within operators. Separate models were fitted for VUA time and modified RACE score as dependent variables. Regression coefficients (β) with 95% confidence intervals (CI) were reported. Because case sequence was log-transformed, β coefficients represent the expected change in the outcome associated with a one-unit increase in the natural logarithm of case sequence and should not be interpreted as the change associated with each additional case.

Inter-rater reliability for video-based assessment was evaluated using the intraclass correlation coefficient (ICC), calculated using a two-way random-effects model with absolute agreement. All statistical tests were two-sided, and a *p*-value < 0.05 was considered statistically significant. Statistical analyses were performed using R 4.5.3 software (R Foundation for Statistical Computing, Vienna, Austria).

## 3. Results

### 3.1. Baseline

A retrospective analysis was conducted on 100 patients who underwent robot-assisted radical prostatectomy and met the study inclusion criteria. Patients were grouped by operating surgeon (Surgeons A–D), with 25 procedures analyzed per surgeon. Baseline demographic, clinical, surgical, and pathological characteristics are summarized in Table 1. No statistically significant differences were observed among the four groups. Median age was comparable across surgeons, ranging from 61 to 64 years (*p* = 0.21). Similarly, no significant differences were found in BMI (*p* = 0.39), preoperative prostate-specific antigen (PSA) levels (*p* = 0.74), or prostate volume (*p* = 0.62). The prevalence of diabetes mellitus ranged from 24% to 52% (*p* = 0.17), while metabolic syndrome was present in 16–28% of patients, without significant intergroup differences (*p* = 0.55). Surgical characteristics were well balanced among the four cohorts. The distribution of nerve-sparing approaches was comparable (*p* = 0.97), with bilateral nerve sparing in 44–56% of cases, unilateral nerve sparing in 28–36% of cases, and no nerve sparing in 16–24% of cases. Pelvic lymph node dissection (PLND) was performed in 40–48% of patients, with no significant differences among surgeons (*p* = 0.92). Final pathological findings were also comparable between groups. The distribution of ISUP grades (1–2 vs. 3–5) did not differ significantly among surgeons (*p* = 0.89). Likewise, pathological stage was similarly distributed across cohorts (*p* = 0.77), with organ-confined disease (pT2) representing the majority of cases. Positive surgical margin rates ranged from 12% to 16% and were comparable among surgeons (*p* = 0.95).

### 3.2. Learning Curve and Technical Performance

**Figure 2:**
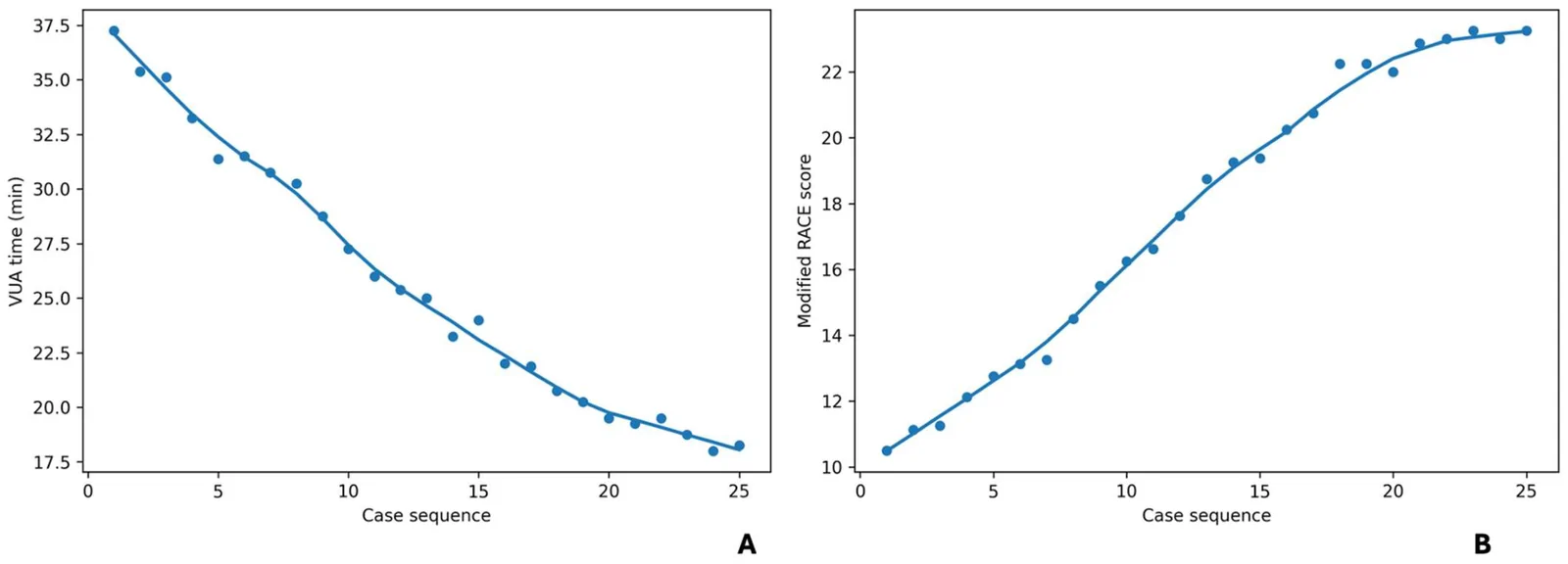
(**A**) Scatter plot with LOWESS smoothing showing the relationship between case sequence and VUA time. Each point represents a single procedure. A progressive decrease in VUA time across consecutive cases is observed. (**B**) Scatter plot with LOWESS smoothing showing the relationship between case sequence and modified RACE score. Each point represents a single vesicourethral anastomosis. A progressive increase in RACE score across consecutive cases is observed.

Technical performance during VUA was evaluated across consecutive cases. Scatter plots of aggregated case sequence data with LOWESS smoothing demonstrated a non-linear relationship between surgical experience and both VUA time and modified RACE score. VUA time decreased across case sequence, with a steeper reduction observed in earlier cases and a more gradual change in later procedures. Conversely, the modified RACE score increased across the case sequence, following a similar non-linear pattern. To quantify these associations, mixed-effects regression models were applied, accounting for clustering at the surgeon level. Case sequence was log-transformed and included as a fixed effect to account for the non-linear structure of the learning curve, while the surgeon was modeled as a random intercept. In this model, increasing surgical experience was significantly associated with a reduction in VUA time (β = −6.93, 95% CI −7.42 to −6.44; *p* < 0.001). Similarly, increasing case sequence was associated with higher modified RACE scores (β = 4.99, 95% CI 4.55 to 5.43; *p* < 0.001). For clinical interpretation, these coefficients correspond to an estimated decrease of approximately 4.8 min in VUA time and an increase of approximately 3.5 points in modified RACE score for each doubling of case sequence. Inter-rater reliability of the modified RACE score was excellent, with an ICC of 0.972, indicating a very high degree of agreement between reviewers. Figure 2 graphically shows our findings.

**Figure 3:**
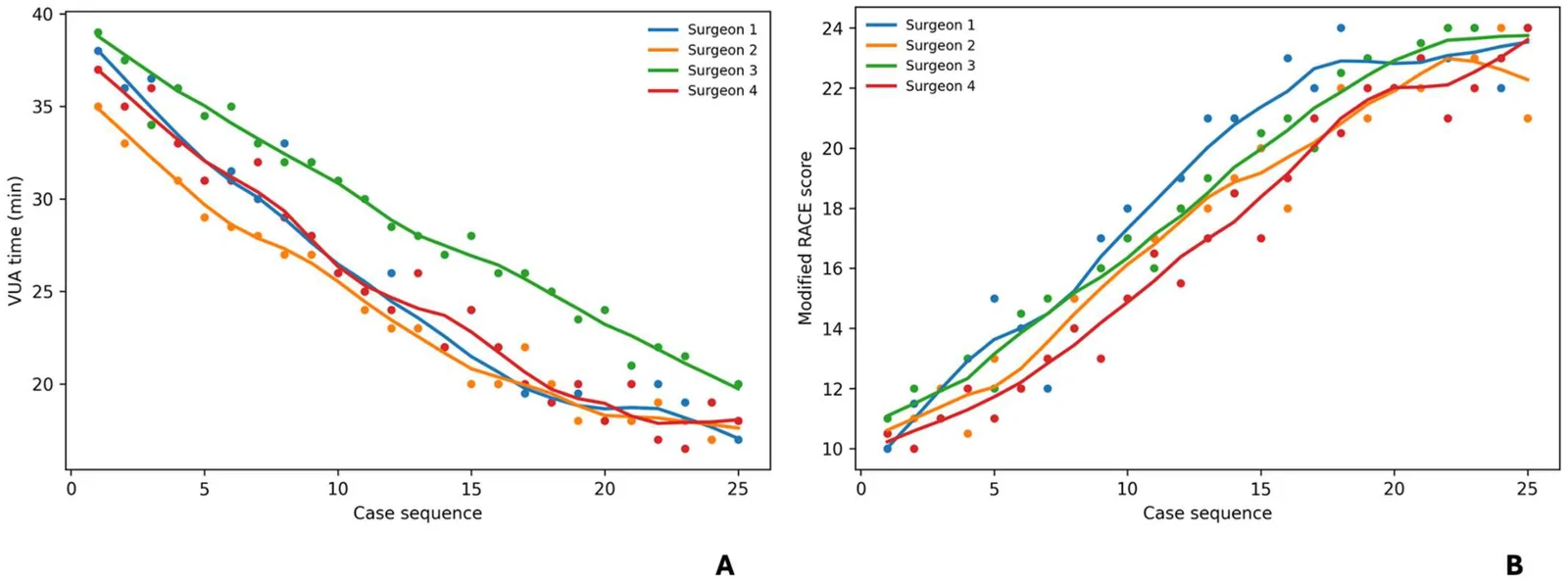
(**A**) Scatter plot with LOWESS smoothing showing VUA time across case sequence, stratified by surgeon. Each point represents a single procedure, and colored lines represent surgeon-specific smoothed trends. (**B**) Scatter plot with LOWESS smoothing showing modified RACE score across case sequence, stratified by surgeon. Each point represents a single vesicourethral anastomosis, and colored lines represent surgeon-specific smoothed trends.

Surgeon-level variability was observed in early cases, with differences in initial VUA time and RACE scores across operators. However, across the case sequence, all surgeons showed consistent changes in both outcomes. When analyzed graphically (Figure 3), individual learning trajectories showed reduced dispersion in later cases.

### 3.3. Anastomosis-Related Intraoperative and Perioperative Outcomes

Median VUA time decreased from 32 min (IQR 29–37) in the early phase to 19 min (IQR 18–20.3) in the late phase (*p* < 0.001). Similarly, the modified RACE score improved from 13 (IQR 11–15) to 22 (IQR 21–23) (*p* < 0.001), reflecting a significant enhancement in anastomotic efficiency and technical quality. A positive intraoperative leak test was observed in 37.5% of cases during the early phase compared with 12.5% in the late phase, showing a statistically significant reduction (*p* = 0.03). The number of additional stitches required to achieve a watertight anastomosis decreased significantly from a median of 1 (IQR 1–2) in the early phase to 0 (IQR 0–1) in the late phase (*p* < 0.001). Similarly, median catheterization time was significantly reduced from 8 days (IQR 7–9) to 6 days (IQR 6–7) (*p* < 0.001). Postoperative urinary leakage occurred in 17.5% of patients in the early phase and 5% in the late phase. Although a reduction was observed, this difference did not reach statistical significance (*p* = 0.16). UC outcomes improved during the early postoperative period. At 30 days, UC was achieved in 15 of 40 patients (37.5%) in the early phase compared with 26 of 40 patients (65%) in the late phase (*p* = 0.025). At 90 days, UC rates were 60% in the early phase and 85% in the late phase (*p* = 0.04). Differences between learning phases diminished over longer follow-up. At 180 days, UC rates were 82.5% and 92.5%, respectively (*p* = 0.48), while at 360 days they were 87.5% and 95% (*p* = 0.43). Table 2 summarizes our findings.

## 4. Discussion

This learning-curve study suggests a measurable improvement in VUA performance from an early to a later phase, with shorter anastomosis times, higher modified RACE technical scores, and fewer corrective stitches. These improvements were associated with shorter catheterization times and improved early UC, while long-term UC rates eventually converged. Several results support a step-specific learning curve for VUA. Procedural efficiency improved significantly, with VUA time decreased from 32 (IQR 29–37) to 19 (IQR 18–20.3) minutes (*p* < 0.001). Technical quality also improved as reflected by an increase in the modified RACE scores from 13 to 22 (*p* < 0.001). Additionally, the need for corrective maneuvers, such as extra stitches, decreased from 1 (1–2) to 0 (0–1) (*p* < 0.001), suggesting more consistent execution of the primary VUA. Regression modeling reinforced these stepwise comparisons. In mixed-effects models using log-transformed case sequence, increasing surgical experience was significantly associated with shorter VUA time (β = −6.93, 95% CI −7.42 to −6.44; *p* < 0.001) and higher modified RACE scores (β = 4.99, 95% CI 4.55 to 5.43; *p* < 0.001). Clinically, the learning curve was most evident in early recovery and perioperative management. Catheter duration decreased from 8 (7–9) to 6 (6–7) days (*p* < 0.001). Postoperative urinary leak decreased numerically from 17.5% to 5%; the difference was not statistically significant, plausibly reflecting insufficient statistical power for a low-frequency endpoint and the sensitivity of ‘leak’ rates to definitions and imaging policy. Thirty-day urinary continence improved significantly from 37.5% to 65% (*p* = 0.025), with a further significant improvement observed at 90 days (60% vs. 85%, *p* = 0.04). By 180 days, continence rates were comparable between groups. This pattern is consistent with the hypothesis that technical refinement primarily influences early functional recovery, whereas long-term continence is likely determined by additional patient- and procedure-related factors [15]. It is, however, important to acknowledge that recovery of urinary continence is multifactorial, influenced not only by VUA quality but also by patient-specific factors, oncologic characteristics, and other surgical steps such as apical dissection and nerve-sparing techniques [16,17,18,19]. A key interpretation of the current data suggests that the rapid early improvements may be due to both general practice effects and the teachability of a well-defined anastomotic technique. This technique features a specific stitch sequence, uniform spacing, and controlled tensioning steps, which help minimize intraoperative variability and reduce the number of real-time decisions. In surgical training, such standardization can reduce the cognitive burden on beginners, allowing them to focus on a limited set of essential robotic skills, such as needle driving, wrist articulation, and depth control. These skills are directly reflected in the progressive improvement of scores over time. This idea is supported by earlier VUA research, where technical improvements were intentionally made to simplify the process. Early in the development of minimally invasive prostatectomy, Hoznek et al. described a two-hemicircumferential running-suture method significantly simplifying the procedure compared with more complex suturing techniques [20]. Later, Yang et al. introduced a simplified continuous single-needle technique, explicitly presenting standardization and simplification as educational benefits rather than mere technical innovations [21]. Within robotic surgery, multiple simplify-and-stabilize strategies show the same educational mechanism. Posterior reconstruction prior to VUA was hypothesized to decrease technical difficulty by stabilizing tissues and relieving tension, and it significantly reduced anastomosis time among surgeons in training [22]. Similarly, barbed or self-cinching sutures maintain approximation after each needle passage, thereby reducing the necessity for repeated tightening and tissue traction. Chapman et al. observed that this method is advantageous for surgeons undergoing training in RARP [23]. The literature further suggests that such “tension-stabilizing” suture strategies reduce the need to readjust tension and can decrease reliance on assistance or constant reassessment of watertight integrity [11,24,25]. Together, these studies support the potential educational value of standardized and reproducible anastomotic techniques. In the present study, however, the absence of a non-standardized comparator group does not allow the specific contribution of standardization to be isolated from the effect of increasing surgical experience. Critically, the educational significance of a standardized VUA becomes even clearer when viewed through the lens of independence. Modern robotic curricula view progression as stepwise entrustment until surgeons can complete a full procedure without mentor intervention [25]. Since VUA is often seen as a high-difficulty reconstructive step, it is typically introduced later in modular training pathways; if the anastomosis itself is standardized and easier to reproduce, it becomes a more feasible candidate for earlier entrustment, helping novices achieve operative independence sooner while still being monitored objectively with a procedure-specific score [26]. This concept is supported by previous structured training experiences. Lovegrove et al. reported a VUA proficiency plateau after approximately 17 cases within a modular RARP training pathway [27]. Similarly, Kato et al. evaluated a standardized institutional training program that divided RARP into six procedural checkpoints, including VUA [28], while Dadashian et al. recently demonstrated in a randomized simulation study that tailored feedback targeting specific technical deficiencies accelerated achievement of a watertight VUA [29]. Together, these findings support structured, step-specific training and objective feedback as complementary strategies for VUA skill acquisition. These findings fit within a body of literature showing that proficiency thresholds vary greatly depending on the endpoints and analytic methods used. Systematic reviews highlight that learning-curve definitions are diverse and often inconsistently applied. Step-specific analyses provide more practical benchmarks. In a large, multi-surgeon CUSUM study focusing on anastomosis time, Nagai et al. reported that approximately 11 cases are needed to complete the initial phase and about 24 cases to complete the consolidation phase for the VUA, supporting the idea that significant VUA improvements occur within the first few dozen cases [30]. Conversely, component-based CUSUM analyses that group VUA with related tasks can produce longer thresholds: Ambinder et al. noted that the VUA required up to 52 cases to stabilize, emphasizing how the length of the learning curve depends on how steps are defined and measured [9]. Time-based improvements comparable to those observed in the present study have also been reported in early experience groups. For instance, Ou et al. reported significant decreases in VUA time as experience increased [31]. In studies of newer platforms, Ramos-Carpinteyro et al. observed a clear early inflection point in VUA time around case 10, supporting the idea that a well-standardized, precisely measured VUA step can show rapid early progress [32]. Procedure-specific technical scoring adds an essential dimension beyond time. RACE was created to objectively assess anastomotic competence, with validation demonstrating clear discrimination across expertise levels [4]. RACE also tracks training progression: among supervised trainees, scores improved significantly over time and were associated with suturing performance [6]. This study extends this paradigm into early independent experience using a modified RACE format, showing a marked improvement from early to late phase and a strong association with case sequence. Finally, the clinical relevance of technical performance is supported by outcome literature. Video-based studies have shown that a surgeon’s technical performance contributes to early continence recovery [15]. Predictive modeling further indicates that VUA technical skill metrics are among the most informative features for continence recovery prediction [33]. These data support the observed pattern: significant improvement at 30 days with convergence by 6–12 months. For novice robotic surgeons, VUA is an ideal training target because it is discrete, repeatable, measurable, and linked to patient-centered outcomes. The present findings suggest that improving VUA quality can shorten catheter duration and accelerate early continence recovery, highly visible outcomes for patients. For counseling during the adoption or early independent phase, the key message is trajectory: early cases may involve longer catheterization and slower early continence recovery, but long-term continence may converge as the learning curve is traversed and as other determinants dominate late recovery. This framing aligns with evidence that technical skill influences early functional recovery and supports transparent, patient-centered counseling during program implementation [15]. In training design, the data supports structured approaches that combine: a standardized technique that reduces decision points and tension-management complexity; an objective, step-specific assessment rather than time alone; and targeted feedback and video review. This is consistent with modular robotic training frameworks, which define progression steps and link entrustment to demonstrable competence rather than case volume alone [26].

## 5. Limitations

This study has several limitations that should be acknowledged. First, its retrospective and single-center design may introduce selection bias and limit the generalizability of the findings. Although all consecutive cases were included, unmeasured confounders cannot be excluded. Second, the sample size is relatively limited, particularly for secondary outcomes such as postoperative urinary leakage, which may reduce the statistical power to detect differences in low-frequency events. Third, the learning curve analysis was based on the chronological sequence of cases, which may not fully account for variability in case complexity or patient-related factors. Although baseline characteristics were comparable across groups, residual confounding cannot be excluded. Fourth, technical performance was assessed through video review using a modified RACE score. While inter-rater agreement was evaluated, the retrospective nature of the assessment and the use of a modified scoring system may introduce measurement bias. Additionally, the analysis focused on a specific step of the procedure rather than the entire surgical workflow. Although this step-specific approach allows for a more precise evaluation of technical progression, it does not capture the full complexity of robot-assisted radical prostatectomy. Finally, functional outcomes such as urinary continence are multifactorial and may be influenced by variables beyond the vesicourethral anastomosis, including patient characteristics and other surgical steps. Therefore, the observed associations between technical performance and clinical outcomes should be interpreted with caution. Prospective, multicenter studies with larger cohorts and standardized assessment tools are warranted to validate these findings and further clarify the role of step-specific training in robotic surgery.

## 6. Conclusions

This study suggests progressive improvements in VUA technical performance among novice robotic surgeons performing the same standardized technique. During the early phases of independent practice, reductions in anastomosis time and improvements in technical quality were observed, along with more favorable perioperative parameters. These technical changes were accompanied by improved early recovery of urinary continence and shorter catheterization time, while long-term continence outcomes appeared comparable across learning phases. The observed relationship between case sequence and both VUA time and modified RACE score supports the concept of a step-specific learning curve. Video-based assessment using a structured scoring system may represent a useful tool for objectively monitoring skill acquisition during robotic training. However, the absence of a non-standardized comparator group does not allow the independent contribution of technique standardization to the observed improvements to be determined. Given the study design, these findings should be interpreted as exploratory and hypothesis-generating. Further prospective and multicenter studies are warranted to confirm these observations.

## Figures and Tables

**Table 1:** Baseline demographic, clinical, and pathological characteristics of patients stratified by surgeon. Continuous variables are reported as median and interquartile range (IQR), and categorical variables as frequencies and percentages. Between-group comparisons were performed using the Kruskal–Wallis test for continuous variables and the chi-square test or Fisher’s exact test for categorical variables, as appropriate.

Variable	Surgeon A (*n* = 25)	Surgeon B (*n* = 25)	Surgeon C (*n* = 25)	Surgeon D (*n* = 25)	*p*-Value
**Age, years, median (IQR)**	61 (58–64)	64 (62–66)	62 (60–66)	62 (60–66)	0.21
**BMI, kg/m^2^, median (IQR)**	26.1 (24.9–26.8)	27.7 (25.3–29.0)	27.8 (25.1–28.2)	26.2 (24.3–28.0)	0.39
**Preoperative PSA, ng/mL, median (IQR)**	7.0 (6.2–8.2)	7.4 (6.6–9.4)	7.6 (6.1–8.6)	7.2 (5.5–8.6)	0.74
**Prostate volume, mL, median (IQR)**	62 (47–71)	53 (35–67)	51 (42–68)	56 (44–67)	0.62
**Diabetes mellitus, *n* (%)**	6 (24%)	12 (48%)	9 (36%)	13 (52%)	0.17
**Metabolic syndrome, *n* (%)**	4 (16%)	7 (28%)	4 (16%)	7 (28%)	0.55
**Nerve-sparing status, *n* (%)**					0.97
**Bilateral**	14 (56%)	11 (44%)	12 (48%)	12 (48%)	
**Unilateral**	7 (28%)	8 (32%)	9 (36%)	9 (36%)	
**None**	4 (16%)	6 (24%)	4 (16%)	4 (16%)	
**PLND, *n* (%)**	10 (40%)	12 (48%)	12 (48%)	12 (48%)	0.92
**ISUP grade (final pathology), *n* (%)**					0.89
**Grade 1–2**	13 (52%)	11 (44%)	12 (48%)	14 (56%)	
**Grade 3–5**	12 (48%)	14 (56%)	13 (52%)	11 (44%)	
**Pathological stage, *n* (%)**					0.77
**pT2**	18 (72%)	21 (84%)	19 (76%)	19 (76%)	
**pT3a**	6 (24%)	3 (12%)	5 (20%)	6 (24%)	
**pT3b**	1 (4%)	1 (4%)	1 (4%)	0 (0%)	
**Positive surgical margins, *n* (%)**	4 (16%)	3 (12%)	4 (16%)	3 (12%)	0.95

**Table 2:** Vesicourethral anastomosis (VUA)-related perioperative and functional outcomes in the early and late phases of the learning curve.

Variable	Early Phase (*n* = 40)	Late Phase (*n* = 40)	*p*-Value
**VUA time, min. (IQR)**	32 (29–37)	19 (18–20.3)	<0.001
**RACE score (IQR)**	13 (11–15)	22 (21–23)	<0.001
**Positive leak test, %**	15 (37.5%)	5 (12.5%)	0.03
**Additional stitches**	1 (1–2)	0 (0–1)	<0.001
**Catheter days, median (IQR)**	8 (7–9)	6 (6–7)	<0.001
**Postoperative urinary leak, *n* (%)**	7 (17.5%)	2 (5%)	0.16
**Continence 30 days, *n* (%)**	15 (37.5%)	26 (65%)	0.025
**Continence 90 days, *n* (%)**	24 (60%)	34 (85%)	0.04
**Continence 180 days, *n* (%)**	33 (82.5%)	37 (92.5%)	0.48
**Continence 360 days, *n* (%)**	35 (87.5%)	38 (95%)	0.43

## Data Availability

The data presented in this study are available upon reasonable request to the corresponding author (due to privacy or ethical restrictions imposed by our institution).

## Supplementary Materials

The following supporting information can be downloaded at: https://www.mdpi.com/article/10.3390/curroncol33090502/s1, Table S1: Modified Robotic Anastomosis Competency Evaluation (RACE) score developed a priori for retrospective blinded video-based assessment of vesicourethral anastomosis. The instrument comprised five technical domains, each scored on a 5-point Likert scale (total score range: 5–25); Video S1: Anastomosis.

## Abbreviations

The following abbreviations are used in this manuscript:

BMI	Body Mass Index
BNS	Bladder Neck Stricture
CI	Confidence Interval
DM	Diabetes Mellitus
HoLEP	Holmium Laser Enucleation of the Prostate
ICC	Intraclass Correlation Coefficient
IQR	Interquartile Range
ISUP	International Society of Urological Pathology
LOWESS	Locally Weighted Scatterplot Smoothing
MeTS	Metabolic Syndrome
PCa	Prostate Cancer
PLND	Pelvic Lymph Node Dissection
PSA	Prostate-Specific Antigen
QoL	Quality of Life
RACE	Robotic Anastomosis Competency Evaluation
RARP	Robot-Assisted Radical Prostatectomy
SD	Standard Deviation
TUIP	Transurethral Incision of the Prostate
TURP	Transurethral Resection of the Prostate
UC	Urinary Continence
VUA	Vesicourethral Anastomosis
