# Supplementary material for: Learning Curve and Video-Based Technical Assessment of a Standardized Vesicourethral Anastomosis in Novice Robotic Surgeons
Source: Curr Oncol. 2026 Aug 25;33(9):502. doi: 10.3390/curroncol33090502 (PMC13605326; doi:10.3390/curroncol33090502)
Supplement: Supplementary file 1 [file curroncol-33-00502-s001.zip › Supplementary Table S1.pdf]

**Domain 1-Needle positioning & driving**

| Score | Definition |
|---|---|
| **1-Poor** | Incorrect needle angles, frequent re-grasping, unsafe or imprecise needle passes requiring repeated corrections |
| **2-Below average** | Inconsistent needle angles with frequent adjustments; acceptable passes achieved only after multiple corrections |
| **3-Adequate** | Generally correct needle trajectory with occasional re-grasping or minor adjustments |
| **4-Good** | Correct needle angles and depth in most passes, with rare need for adjustment |
| **5-Excellent** | Consistently precise needle angles and depth, smooth needle driving without re-grasping |

**Domain 2-Suture handling**

| Score | Definition |
|---|---|
| **1-Poor** | Loss of suture tension, frequent entanglement, inefficient or interrupted movements |
| **2-Below average** | Intermittent loss of tension or minor entanglement requiring correction |
| **3-Adequate** | Acceptable suture handling with minor inefficiencies |
| **4-Good** | Consistent suture control with efficient movements and rare minor inefficiencies |
| **5-Excellent** | Continuous tension control, fluid and efficient suture handling throughout |

**Domain 3-Tissue handling & respect**

| Score | Definition |
|---|---|
| **1-Poor** | Excessive traction, tissue trauma, or tearing |
| **2-Below average** | Occasional excessive tension causing tissue distortion without frank injury |
| **3-Adequate** | Generally appropriate tissue handling with minor tension issues |
| **4-Good** | Gentle tissue handling with minimal unnecessary traction |

| Score | Definition |
|---|---|
| **5-Excellent** | Consistently gentle handling with optimal tissue preservation |

**Domain 4-Symmetry & tissue approximation**

| Score | Definition |
|---|---|
| **1-Poor** | Asymmetric bites, poor alignment, inadequate mucosal apposition |
| **2-Below average** | Frequent asymmetry or uneven spacing requiring correction |
| **3-Adequate** | Mostly symmetric bites with minor misalignment |
| **4-Good** | Symmetric and well-spaced bites with minimal irregularity |
| **5-Excellent** | Perfect symmetry, uniform spacing, optimal mucosal apposition |

**Domain 5-Overall anastomotic quality**

| Score | Definition |
|---|---|
| **1-Poor** | Interrupted flow, multiple corrections required, low confidence execution |
| **2-Below average** | Frequent hesitations and corrections, suboptimal flow |
| **3-Adequate** | Completed with minor issues but acceptable final result |
| **4-Good** | Smooth execution with minimal corrections |
| **5-Excellent** | Confident, fluid execution resulting in a watertight anastomosis |

Supplementary Table S1. Modified Robotic Anastomosis Competency Evaluation (RACE) score developed *a priori* for retrospective blinded video-based assessment of vesicourethral anastomosis. The instrument comprised five technical domains, each scored on a 5-point Likert scale (total score range: 5–25).
